# Psychological Well-Being during the COVID-19 Lockdown: Labour Market and Gender Implications

**DOI:** 10.1007/s11482-022-10113-4

**Published:** 2022-12-23

**Authors:** Israel Escudero-Castillo, Fco. Javier Mato-Díaz, Ana Rodríguez-Alvarez

**Affiliations:** 1 Department of Applied Economics, University of Oviedo, Oviedo, Spain; 2 Department of Economics, University of Oviedo, Oviedo, Spain

**Keywords:** COVID-19, Well-being, Labour situation, Mental health

## Abstract

In the Spring of 2020, a great number of countries introduced different restrictive measures in order to cope with the COVID-19 pandemic. This article examines the labour market transitions of individuals brought about by some of those measures, and the effect of such transitions on psychological well-being. The fact that it has been possible to distinguish between unemployment transitions before the pandemic began and those resulting from the lockdowns is worth highlighting. Evidence is provided showing that unemployment due to the lockdown had a greater negative impact on psychological well-being than furloughs and teleworking. Gender differences confirm that women experienced greater adverse effects as compared to men. Specifically, women working at home exhibited greater negative effects when compared with those on furlough, probably due to a combination of work disruption and increased family obligations. Finally, on the contrary to men, women living in areas with more rigorous restrictions show a reduced probability of worse PWB when compared to those residing in areas without restrictions. This finding suggests that women are willing to sacrifice freedom of movement as long as restrictions protect their at-risk relatives.

## Introduction

The COVID-19 pandemic created an unprecedented economic situation that had a devastating impact on the world economy, with rigorous restrictions heavily affecting freedom of movement (Shek, 2021). During the first half of 2020 economic lockdowns were widespread and led to workplace closures. Labour market disruption led to many people becoming unemployed, while others were furloughed or started teleworking. Other workers maintained business as usual under the supervision of health authorities. According to the International Labour Organisation, 93 per cent of the world’s workers resided in countries with some sort of workplace closure measures in place during the first half of 2020 (International Labour Organization, 2020).

The impact of the COVID-19 crisis disproportionately affected female workers. Jobs held by women were at greater risk than those held by men due to the unique consequences of the lockdowns and freedom of movement restrictions on the service sector. For example, sectors with higher-than-average female employment, such as health and social services, were under extraordinary stress due to the pandemic. Also, it is likely that the additional burden of care work in the family affected women more than men.

In order to mitigate the effects of the pandemic, different countries implemented a range of measures in order to control its spread. While some European countries such as Spain or Italy ceased all non-essential activity, others, such as the USA reacted later and in a less centralized manner. Different degrees and timing of restrictions may have shaped the way in which measures have affected working situations and thus, the psychological well-being (PWB)^1^ of the population. The aim of this study is to analyse the changes in the employment situations of individuals due to these restrictions (unemployment, teleworking or furloughs) and the effect of such changes on PWB.

Becoming unemployed may bring about loss of income, social relationships and other advantages of having a job such as objectives and purposes that transcend the individual, ascription to status and obligation to maintain a certain level of activity (Jahoda, 1982). The transition from employment to unemployment or from working in the workplace to telecommuting may therefore have affected the mental well-being of workers. Controlling for, amongst other variables, economic sector and the different requirements regarding workplace closures and household conditions, the events of 2020 created favourable conditions for amplifying the literature that relates PWB with the labour market consequences of the pandemic. That is the objective of this article.

Thus, we have designed an ad-hoc questionnaire in order to obtain information about working situations, well-being status and other socio-demographic characteristics. The survey allows us to distinguish between how people were employed prior to the onset of the COVID-19 pandemic and how their employment situation changed during confinement. Although most of the sample is made up of Spanish workers, given that the degree of restrictions on mobility depended on the measures imposed by governments, the survey was extended to the population of other countries during the same period in the hope that cross-country comparison would make it possible to capture how different levels of mobility restrictions could be affecting PWB. Finally, it is worth highlighting that the restrictions created exceptional circumstances in the labour market that, in turn, offer a unique opportunity to analyse the influence of employment situation on PWB. For example, many countries reacted to the COVID-19 crisis by making widespread use of furloughs.

This research contributes to the existing literature in two areas. First, sudden changes in work-related situations serve as external shocks that minimize the bidirectionality problems common to this type of studies. In general, the existing literature that relates employment situation with mental health must tackle the bidirectionality between these two variables. Specifically, when studying whether unemployment can affect PWB, the hypothetical effect that mental health has on the probability of becoming or remaining unemployed should be isolated in order to not bias the results. A lack of control of bidirectionality could lead to overestimating the effect of the unemployment situation on PWB, since part of this impact may be due to a greater probability of being unemployed for people with worse PWB. Thus, the main contribution of this article comes from being able to distinguish, among unemployed workers, between those who were already unemployed and those who became jobless as a direct result of the pandemic. Second, this research aims to expand the knowledge provided by the existing literature on the evolution of mental health during the pandemic and how this evolution could be dependent on factors such as the person's employment situation or the degree of restrictions on mobility.

The structure of the article is as follows: in the next section, the literature discussing the relationship between PWB and employment situation during pandemics is reviewed. Then, the empirical model and the data are presented in sections three and four. In the fifth section we analyse the results, and a final section is devoted to conclusions and discussion.

## Literature Review

Although the lockdown due to the COVID-19 pandemic is very recent, there is some research that has attempted to analyse its detrimental effects on PWB. Since the beginning of the pandemic, this literature has focused on the origins of worse PWB, concluding that three main factors are causing it: permanent exposure to a fear of contagion of oneself or a family member (Cao et al, 2020; Li et al, 2020), restrictions imposed by different governments (Brooks et al., 2020) and overexposure to news related to the pandemic (González-Sanguino et al, 2020; Yao, 2020). Furthermore, this reduction in PWB does not seem to be uniform, but instead accentuated among certain circumstances. Thus, groups such as women, young people and immigrants seem more prone to this deterioration (García-Álvarez et al, 2020; Pieh et al, 2020; Proto & Quintana-Domeque, 2021). Additionally, this relationship may be affected by external factors such as access to information about the pandemic, friendship quality, trust in public institutions such as the government, judiciary or mass media, or the type of confinement (self-imposed vs. lockdown) (Bittmann, 2022; Lu et al, 2021; Ye et al., 2022).

One of the most interesting consequences of the COVID-19 pandemic is its effects on labour markets. Employment situation changes such as moving from the normal workplace into teleworking, entering a furlough scheme or becoming unemployed, could also be related to PWB. There is evidence available about the impact of these three most significant employment changes. Regarding the loss of employment, recent research carried out during the pandemic for the USA (Yao & Wu, 2021), Spain (Escudero-Castillo et al, 2021; González-Sanguino et al, 2020), Japan (Ikeda et al, 2022) and South Africa (Posel et al, 2021) highlights that job loss during the disruption caused by the pandemic has negatively affected psychological well-being and quality of life. Additionally, these findings about the loss of employment are in line with research carried out before the pandemic (Clark et al, 2001; Cygan-Rehm et al, 2017; Paul & Moser, 2009).

Recent research into the way in which different work transitions are related to different degrees of PWB confirm that transitions from employment into joblessness are associated with worsening PWB, while transitions from unemployment into work are related to improvements in it (Arya et al, 2021). Several authors have considered gender to be one of the most interesting variables when it comes to analyzing how work transitions affect mental health, finding that men seem to be more sensitive to the negative effects of unemployment (Strandh et al, 2013). This gender difference is especially accentuated in countries where the role of men is more associated with the labour market than that of women. Gender stereotypes cause women’s roles to be relatively more connected to caring activities outside the labour market and, thus, unemployment may have a moderated effect on them. Likewise, this greater labour market attachment of men is the cause of PWB improvements related to transitions from unemployment into work also being superior among men when compared to women (Chung & Hahn, 2021; Huber et al, 2011).

The relationship between teleworking and PWB seems less clear. Moens et al. (2021) find contradictory effects in this matter: teleworking promotes greater efficiency and a lower risk of burnout while at the same time reducing probability of job promotion and weakening ties with colleagues and employers. Furthermore, people obliged to work from home during the lockdown could have changed their perceptions about work and started considering their jobs less meaningful, which in turn may negatively affect their engagement at work, a finding made among health workers by Wijngaards et al. (2022). Another occupation that has been strongly affected by teleworking during the pandemic was that of academics. In this sense, Ugwu et al. (2022) found that teleworking during the closure of universities in Nigeria was related to lower work-life-balance among university teaching staff with high levels of work engagement. In the literature, we can find other examples of ambivalence regarding the effects of teleworking on PWB: some studies find positive effects (Kossek et al, 2006; Tavares, 2017), while others find negative ones (Escudero-Castillo et al, 2021; Mann & Holdsworth, 2003; Song & Gao, 2020). However, these contradictory effects of teleworking could be more related to different degrees of organizational support (in terms of regular communication or suitable workloads), peer support or levels of conflict between work and family obligations rather than the teleworking situation itself (Oakman et al, 2020).

Research on transitions from employment into furloughs is scarce. However, the work by Posel et al. (2021) concludes that previously employed workers who became unemployed or furloughed during the pandemic were more prone to depression than those who retained their jobs. These authors differentiated between paid and unpaid furloughs, concluding that the possible protective effect of a furlough scheme, due to workers knowing of the existence of a job to which they will return when the situation improves, disappears in the absence of income.

All in all, additional evidence is necessary about the consequences of employment situation changes for PWB. The lockdowns due to COVID-19 may facilitate such analyses since they prompted unexpected unemployment situations, as well as sudden growth of teleworking around the world. There is already evidence about the effect of the pandemic on the PWB of individuals, for example, in regard to greater anxiety and stress (González-Sanguino et al, 2020). Generally speaking, the situation can be said to be leading to the appearance of relatively serious mental health problems, the scope of which has not yet been clearly defined.

In this context, the effect of teleworking, furloughs and the different effects of changes in work situations on the PWB of men and women are the topics to be addressed in this paper. The specific questions to be answered are the following: What is the relationship between an employment transition into joblessness and PWB? How much of an effect on PWB does movement from the usual workplace into teleworking have? Does PWB, *ceteris paribus*, vary as a result of differences in the degree of restrictions on freedom of movement? And finally, are there significant gender differences in these effects?

## Data

For the purposes of this research, a database was created by means of designing a specific questionnaire and conducting an online survey of 1,165 workers. This survey was completed during the period April 11^th^ to May 7^th^, 2020, at the peak of the first wave of the COVID-19 pandemic during which the strictest confinements were occurring in Spain, Europe and across large parts of the world. Although the majority of respondents were working Spanish residents, the survey was supplemented with people living in other countries^2^ which had different restrictive measures in order to evaluate how differing restrictions during the lockdown may influence PWB. Two conditions were established for respondents: being involved in the labour market and having had at least one job during the previous 12 months.

In order to tackle several standard issues typical of online information methods (Baltar & Brunet, 2012) such as self-selection bias or lack of representativeness, elevation coefficients were used. In this regard, in addition to the criteria of gender, three age groups and three educational levels and quota sampling were applied to the group of respondents. The combination of the criterion variables resulted in 18 subsamples or quotas.^3^

In Table 1 the variables used in the model are presented. In order to capture workers’ psychological well-being, we have used Goldberg’s General Health Questionnaire (Goldberg, 1967) (12-items version). This questionnaire is a self-administered originally aimed at detecting non-psychotic psychiatric illnesses among general practice patients (Goldberg & Blackwell, 1970). Its original version consisted of 60 items, with later shorter versions consisting of 28 (Goldberg & Hillier, 1979) and 12 items (Goldberg et al, 1997) being developed. The version administered in this investigation was the 12-item reduced version, which is one of the most widely used screening instruments (Chung & Hahn, 2021; Clark, 2021; Sánchez-López & Dresch, 2008) and included in surveys such as the Spanish National Health Survey or the British Household Survey Panel. Furthermore, studies carried out for the analysis of the internal consistency of this test offer Cronbach's alpha coefficients of 0.76 (Sánchez-López & Dresch, 2008) or 0.87 (Montazeri et al., 2003), representing satisfactory results and, therefore, making it a suitable instrument for evaluation of PWB and detection of non-psychotic psychiatric disorders. The treatment of the components of the GHQ-12 was based upon an initial calculation for each respondent of the mean of the results of the 12 items. Then, this mean was recalculated as an ordinal variable with three values: low, medium and high risk.

**Table 1:** Variables included in the analysis

Variable	Variable description
Risk of suffering PWB problems	Categorical variable comprising low, medium and high levels of risk
Working situation	Categorical variable covering whether the person was unemployed before the lockdown; working before the lockdown but becoming unemployed during it; on furlough due to the pandemic; on leave; teleworking; or working in the usual pre-pandemic workplace
Economic sector	Categorical variable comprising the primary, secondary and tertiary sectors
Occupational group	Categorical variable comprising directors and managers; scientific and intellectual professionals; support professionals; accounting and administrative employees; catering, security, vendors and other lower-skilled workers
Years of work experience	Continuous variable
Income	Categorical variable comprised of people with an income of 800€ to 1200€; between 1201€ and 2000€; and above 2000€
Restrictions during confinement	Categorical variable including if people could go outside without any restrictions; if they could go outside but only for a limited time; or if they could go outside only for specific matters such as buying food or work-related reasons
Confinement in Spain	Dichotomous variable showing whether the respondent is confined in Spain or elsewhere
Gender	Dichotomous variable
Age	Continuous variable
Education level	Categorical variable including low, medium and university levels
Disability	Dichotomous variable illustrating if the person has a disability
Marital status	Categorical variable showing if the person is married, separated, divorced, single or widowed
Household members at risk of COVID-19	Dichotomous variable covering whether the person lives with someone at risk of infection by COVID-19
Spain as country of birth	Dichotomous variable covering whether the person was born in Spain
Number of people confined in the household	Categorical variable showing the number of people in the household, from one to “five or more” individuals
Minors in the household	Dichotomous variable covering whether minors live in the household
Dwelling with a patio	Dichotomous variable that covers whether people can use a patio or garden
M^2^ per capita in the dwelling	Continuous variable aimed at considering the amount of living space

number of observations = 1165 (Men = 481; Women = 684)

Given the multidimensionality of the GHQ-12, the test can be separated into different factors. Some authors (Werneke et al, 2000) conclude there are two factors: anxiety and depression on the one hand, and social dysfunction on the other. Others, however, conclude that a third factor should be added to this structure: loss of confidence (Graetz, 1991). In this regard, Romppel et al. (2013) conclude that unidimensional interpretation offers a useful screening measure for mental distress. Thus, although using different dimensions could be useful for measuring specific factors of psychological distress, it “does not offer many practical advantages in differentiating clinical groups or identifying association with clinical or health-related quality of life variables” (Gao et al, 2004). Because of this, and given that the main aim of this paper is to observe how the pandemic may be affecting psychological well-being, we have not deemed it necessary to look at the impact on the separate dimensions.

Regarding the independent variable around which the research is centered (working situation), respondents were given six mutually exclusive options: employed, unemployed before the lockdown, becoming unemployed during the lockdown, furloughed due to lockdown, on leave for other reasons, and teleworking.

## Method

The ordered probit model is constructed around a latent regression as follows:

1 $${Y}^{*}={X}^{^{\prime}}\beta +\varepsilon$$

where Y^*^is an unobserved dependent variable, *X* is a vector of explanatory variables, *β* is a set of parameters in the model and *ε* is a random term normally distributed.^4^ What is generally observed instead of Y^*^ is the categorical variable *Y*, which can be represented as:

2 $$\begin{array}{l}\begin{array}{cc}Y=0&if\;Y^\ast\leq\mu_1\end{array},\\\begin{array}{cc}Y=1&if\;\mu_1\leq Y^\ast\leq\mu_2\end{array},\\\begin{array}{l}\begin{array}{cc}Y=2&if\;\mu_2\leq Y^\ast\leq\mu_3\end{array},\\\begin{array}{c}\vdots\\\begin{array}{cc}Y=M&if\;\mu_M\leq Y^\ast\end{array},\end{array}\end{array}\end{array}$$

where the cutpoints, *μ*s, are unknown parameters to be estimated along with *β* in the model, and *M* are the possible outcomes for *Y*. After normalising the mean and variance of *ε* to zero and one, the probabilities associated with the alternative values that the observed variable *Y* can have can thus be represented as:

3 $$\begin{array}{l}Prob\left(Y=0|X\right)=\Phi \left({\mu }_{1}-{X}^{^{\prime}}\beta \right),\\ Prob\left(Y=1|X\right)=\Phi \left({\mu }_{2}-{X}^{^{\prime}}\beta \right)-\Phi \left({\mu }_{1}-{X}^{^{\prime}}\beta \right),\\ \begin{array}{l}Prob\left(Y=2|X\right)=\Phi \left({\mu }_{3}-{X}^{^{\prime}}\beta \right)-\Phi \left({\mu }_{2}-{X}^{^{\prime}}\beta \right),\\ \begin{array}{c}\vdots \\ Prob\left(Y=M|X\right)=1-\Phi \left({\mu }_{M}-{X}^{^{\prime}}\beta \right),\end{array}\end{array}\end{array}$$

where *Φ* represents the cumulative distribution function of a standard normal distribution. For all the probabilities to be positive, the *μ*s should fulfil:

4 $$0<{\mu }_{1}<{\mu }_{2}<\dots <{\mu }_{M}.$$

The non-linear model described before can be estimated through a maximum likelihood approach. In this study we follow this model using three cutpoints, which result in three risks of suffering mental disorder that in this text we have identified as low, medium and high risk.

## Results

The article presents three types of analyses. The first analysis makes use of descriptive statistics. The distribution of the risk of suffering a PWB problem across all the independent and control variables is exhibited in Table 2. Differences in the proportion of people at high risk can be seen to be highly correlated with working situation. The proportion is notable among the group who became unemployed during the lockdown (28.0%), while it is small among those teleworking or working in the usual workplace. The group made up of those who were previously unemployed, and workers furloughed or on leave, show intermediate proportions of people at high risk. Regarding differences by gender, a larger proportion of women show high risk of PWB problems (10.67%), when compared with men (7.90%).

**Table 2:** Distribution of the risk of suffering a PWB problem

	High risk	Medium risk	Low risk
Working situation (%)			
Unemployed (out of work before the lockdown)	16.67	53.85	29.49
Becoming unemployed (out of work during the lockdown)	28.00	54.00	18.00
Furloughed due to the lockdown	15.17	59.55	25.28
On leave for other reasons	14.29	50.00	35.71
Teleworking	7.02	59.70	33.27
Working in the usual workplace	4.71	60.87	34.42
Economic sector (%)			
Primary	3.45	55.17	41.38
Secondary	8.48	55.15	36.36
Tertiary	9.89	59.73	30.38
Occupational group (%)			
Directors and managers	13.16	60.53	26.32
Scientific and intellectual professionals	7.41	58.10	34.48
Support professionals	10.91	55.76	33.33
Accounting and administrative employees	10.34	64.66	25.00
Catering and vendors	15.29	59.87	24.84
Other lower-skilled workers	8.65	60.58	30.77
Years of work experience (average)	16.30	19.28	21.18
Income (%)			
Low level	15.00	59.38	25.62
Medium level	9.68	59.57	30.75
High level	4.84	57.53	37.63
Restrictions during confinement (%)			
Without restrictions	11.11	55.56	33.33
Medium level of restrictions	0.00	75.00	25.00
High level of restrictions	9.51	59.02	31.47
Confinement in Spain (%)			
Yes	9.81	58.76	31.43
No	6.96	60.87	32.17
Gender (%)			
Men	7.90	55.93	36.17
Women	10.67	61.11	28.22
Age (average)	41.31	44.02	45.95
Educational level (%)			
Low level	14.46	60.24	25.30
Medium level	10.84	59.79	29.37
High level	8.54	58.54	32.91
Disability (%)			
Yes	9.50	58.79	31.71
No	11.11	61.11	27.78
Marital status (%)			
Married	8.75	59.35	31.90
Separated or divorced	9.84	56.56	33.61
Single	10.54	59.19	30.27
Widowed	9.09	63.64	27.27
Household members at risk of COVID-19 (%)			
Yes	11.90	58.73	29.37
No	8.31	59.09	32.60
Spain as country of birth (%)			
Yes	9.96	59.09	30.95
No	6.11	58.02	35.88
Number of people confined in the household (av.)	2.79	2.68	2.63
Minors in the household (%)			
Yes	10.56	59.10	30.34
No	8.89	58.89	32.22
Dwelling with a patio (%)			
Yes	7.44	59.50	33.06
No	10.12	58.87	31.01
M^2^ per capita in dwelling (average)	35.59	36.38	40.03

Table 3 includes the distribution of all variables in the analysis by gender. The most noticeable differences between men and women are found with regard to educational levels (women are more educated than men), income (men make more money than women) and the economic sector to which the current or last job pertains (there are more women than men in the service sector, and fewer in the primary and secondary sectors). Employment situation differences by gender are statistically significant with respect to “working in the usual workplace” (Diff: 0.05; p = 0.049; CI: -0.0004, 0.0999) and, to a lesser extent, “on leave for other reasons” (Diff: -0.019; p = 0.088; CI: -0.0396, 0.0018). The remaining employment situation differences by gender are not statistically different from zero.

**Table 3:** Distribution of variables by gender

	Total sample	Men	Women
Working situation (%)			
Unemployed (out of work before the lockdown)	6.70	6.65	6.73
Becoming unemployed (out of work during the lockdown)	4.29	4.37	4.24
Furloughed due to the lockdown	15.28	13.93	16.23
On leave for other reasons	3.61	2.49	4.39
Teleworking	46.44	45.95	46.78
Working in the usual workplace	23.69	26.61	21.64
Economic sector (%)			
Primary	2.49	3.53	1.75
Secondary	14.16	22.66	8.19
Tertiary	83.35	73.80	90.06
Occupational group (%)			
Directors and managers	3.28	5.00	2.06
Scientific and intellectual professionals	50.00	44.17	54.12
Support professionals	14.22	17.71	11.76
Accounting and administrative employees	10.00	6.25	12.65
Catering and vendors	13.53	11.04	15.29
Other lower-skilled workers	8.97	15.83	4.12
Years of work experience (average)	19.59	20.65	18.85
Income (%)			
Low level	27.66	20.92	32.40
Medium level	40.19	40.38	40.06
High level	32.15	38.70	27.54
Restrictions during confinement (%)			
Without restrictions	3.09	4.37	2.19
Medium level of restrictions	0.34	0.21	0.44
High level of restrictions	96.57	95.43	97.37
Confinement in Spain			
Yes	90.13	88.36	91.37
No	9.87	11.64	8.63
Age (average)	44.37	44.32	44.40
Educational level (%)			
Low level	7.12	11.85	3.80
Medium level	24.55	29.73	20.91
High level	68.33	58.42	75.29
Disability (%)	3.10	3.13	3.08
Marital status (%)			
Married	50.17	54.58	47.07
Separated or divorced	10.50	10.00	10.85
Single	38.38	34.58	41.06
Widowed	0.95	0.83	1.03
Household members at risk of COVID-19 (%)			
Yes	33.91	32.85	34.65
No	66.09	67.15	65.35
Spain as country of birth (%)			
Yes	88.76	87.53	89.62
No	11.24	12.47	10.38
Number of people confined in the household (avg.)	2.68	2.77	2.62
Minors in the household (%)			
Yes	38.20	41.58	35.82
No	61.80	58.42	64.18
Dwelling with a patio (%)			
Yes	20.84	20.71	20.94
No	79.16	79.29	79.06
M^2^ per capita in dwelling (average)	37.46	37.53	37.41

In line with what has been explained in Sect. 4, the second analysis uses ordered probit models to examine the relationship between the varying work situations during the lockdown and risks of suffering a PWB problem. Results are presented in Table 4, which shows results for the whole sample, as well as separate analyses for men and women. Regarding the full sample results, the work-related situation variable seems to be related to PWB. The three situations that reflect labour market disruption due to COVID-19 show positive coefficients significantly different from zero. Teleworking, being furloughed, and especially being unemployed due to the lockdown are associated with greater risk of suffering from a mental health problem when compared with the reference situation of working in the usual workplace. This result is also observed in the analyses carried out separately for men and women. Having been unemployed before the lockdown also shows a significant positive coefficient, but for men only. In all three models the highest risk corresponds to those working before lockdowns who became jobless due to the disruption.

**Table 4:** Ordered probit model results

	Total sample			Men			Women		
	Coef.		P >|t|	Coef.		P >|t|	Coef.		P >|t|
Working situation (ref: working in the usual workplace)									
Unemployed (out of work before the lockdown)	0.355		0.163	0.912	***	0.004	–0.196		0.474
Becoming unemployed (out of work during the lockdown)	1.625	***	0.000	1.058	***	0.006	2.604	***	0.000
Furloughed due to the lockdown	0.882	***	0.000	1.028	***	0.000	0.452	*	0.071
On leave for other reasons	0.197		0.626	0.589		0.264	–0.324		0.521
Teleworking	0.727	***	0.003	0.389	*	0.088	0.679	***	0.008
Economic sector (ref: secondary)									
Primary	–1.106	**	0.014	–1.458	***	0.004	0.037		0.949
Tertiary	–0.320		0.155	–0.546	***	0.010	0.202		0.643
Occupational group (ref: Directors and managers)									
Scientific and intellectual professionals	–0.713	***	0.007	–0.328		0.317	–0.970	**	0.012
Support professionals	–0.630	**	0.026	–0.245		0.479	–1.119	**	0.012
Accounting and administrative employees	–0.675	**	0.032	–0.101		0.765	–0.922	**	0.039
Catering and vendors	–0.288		0.347	–0.489		0.218	–0.304		0.475
Other lower–skilled workers	–0.334		0.305	–0.414		0.262	–0.971		0.109
Years of work experience (log continuous var.)	–0.309	**	0.050	0.193		0.401	–0.499	**	0.013
Income (ref: low level)									
Medium level	–0.076		0.692	–0.367		0.123	0.324		0.173
High level	–0.050		0.834	–0.414		0.165	0.364		0.159
Restrictions during confinement (ref: without restrictions)									
Medium level of restrictions	–0.582		0.424	1.493	***	0.001	–1.387		0.172
High level of restrictions	–0.311		0.446	0.383		0.412	–1.192	*	0.095
Confinement in Spain (ref: no)	–0.072		0.824	–0.086		0.849	–0.114		0.749
Gender (ref: men)									
Women	0.381	**	0.014						
Age	–0.023	*	0.051	–0.039	**	0.024	–0.024		0.125
Education level (ref: low level)									
Medium level	–0.058		0.753	–0.125		0.559	–0.227		0.361
High level	–0.121		0.554	–0.024		0.927	–0.618	**	0.023
Disability (ref: no)	0.363		0.309	0.596		0.383	0.212		0.393
Marital status (ref: married)									
Separated or divorced	–0.201		0.535	0.396		0.223	–0.693	**	0.039
Single	–1.124	***	0.000	–0.958	***	0.000	–0.934	***	0.000
Widowed	–0.641		0.429	–1.309		0.298	0.399		0.305
Household members at risk of COVID–19 (ref: No)	0.156		0.308	–0.094		0.612	0.520	**	0.012
Spain as country of birth (ref: no)	0.163		0.605	–0.324		0.380	0.709	**	0.047
Number of people confined in the household (ref: person living alone)									
Two people	–0.970	***	0.001	–0.462		0.180	–1.154	***	0.001
Three people	–0.929	**	0.019	0.420		0.325	–1.680	***	0.001
Four people	–0.826	**	0.026	0.275		0.559	–1.381	***	0.002
Five or more	–1.011	**	0.045	0.365		0.546	–2.023	***	0.000
Minors in the household (ref: no)	–0.209		0.381	–0.746	***	0.007	0.472	**	0.027
Dwelling with a patio (ref: no)	–0.244		0.167	–0.316		0.152	–0.061		0.777
M2 per capita in dwelling (continuous variable)	–0.010	*	0.090	0.001		0.936	–0.013		0.123
Cut1	–4.577	***	0.000	–2.850	***	0.002	–6.156	***	0.000
Cut2	–2.511	***	0.003	–0.768		0.404	–3.7476	***	0.003

^***^ Statistical significance: 1%; ** Statistical significance: 5%; * Statistical significance: 10%

The results assigning the lowest risk to the reference category stand out. Continuing to carry out the job in the usual workplace implies some risks due to potential exposure to COVID-19 during work and/or commutes. However, and although there is no information available on the health and safety measures that firms may have introduced to mitigate the pandemic, when compared to the PWB of people in alternative situations, a clear picture emerges that business-as-usual is the least harmful situation.

The probit models show interesting results in the three spheres of the world of work, confinement conditions and personal and family circumstances. Firstly, regarding work-related variables, the economic sector matters for men, but appears to be not significant for women. Furthermore, men in the primary and secondary sectors show lower risk than those in the tertiary sector. The primary sector reduces risks for the whole sample, perhaps due to limited impact of restrictions on its activities. Notably, occupational group matters for the whole sample and for women but is not significant for men. Thus, women who work as professionals or administrative employees have a reduced risk when compared to women who are directors or managers. Work experience reduces risks for the whole sample and for women in particular but is not significant for men. Additionally, income does not make a difference.

Secondly, in regard to the sphere of confinement conditions, a medium level of restrictions increases the probability of suffering mental disorders when compared with the absence of restrictions, but this result is valid for men only. Curiously enough, a high level of restrictions reduces the risk of worse PWB, but this result is valid only for women. Therefore, the models suggest that limits imposed by authorities in order to mitigate the pandemic benefit the PWB of women, while at the same time reducing that of men.

Thirdly, in the sphere of personal and family circumstances, the most noticeable results have to do with marital status and household composition. With respect to marital status, people who are single show a reduced risk of PWB distress when compared to married respondents. Furthermore, being divorced reduces the risk for women, but not for men. With regard to household composition, the number of people confined in the household is clearly a significant factor. The higher the number of cohabitants, the lower the associated risk of distress, with people living alone suffering the highest risk. Additionally, the presence of minors in the household appears to have differing influences on men and women. While minors reduce the probability of PWB distress for men, they have the opposite effect on women. Finally, the company of someone at risk of COVID-19 negatively affects women but not men.

The third and final type of results comes from estimating marginal effects of the ordered probit models. Marginal effects shown in Table 5 represent changes in the probability of obtaining a point score compatible with a low, medium or high risk of reduction in PWB. With the aim of this study in mind, these effects are shown with regard to work-related situations. The results indicate that transition from employment into joblessness stands out as the change with the worst consequences for the PWB of men and women. Job loss significantly reduces the probability of men and women belonging to the low-risk population, by 30.5% and 34.1% respectively. Likewise, becoming unemployed due to lockdown constraints increases the probability of being in the high-risk group by 14.7% for men and 63.6% for women.

**Table 5:** Marginal effects by work-related situations

		Total Sample			Men			Women		
		Coef.		P >|t|	Coef.		P >|t|	Coef.		P >|t|
Unemployed (out of work before the lockdown)										
Low Risk	–0.115			0.160	–0.270	***	0.002	0.056		0.475
Medium Risk	0.077			0.159	0.154	***	0.001	–0.037		0.470
High Risk	0.038			0.190	0.116	**	0.033	–0.018		0.495
Becoming unemployed due to the lockdown										
Low Risk	–0.394		***	0.000	–0.305	***	0.001	–0.341	***	0.000
Medium Risk	0.060			0.457	0.159	***	0.000	–0.294	**	0.011
High Risk	0.334		***	0.001	0.147	*	0.058	0.636	***	0.000
Furloughed due to the lockdown										
Low Risk	–0.261		***	0.000	–0.298	***	0.000	–0.115	*	0.070
Medium Risk	0.131		***	0.003	0.158	***	0.000	0.053		0.118
High Risk	0.130		***	0.001	0.140	***	0.005	0.062	*	0.085
On leave for other reasons										
Low Risk	–0.065			0.622	–0.183		0.237	0.093		0.530
Medium Risk	0.046			0.609	0.121		0.161	–0.065		0.560
High Risk	0.019			0.654	0.061		0.388	–0.028		0.464
Teleworking										
Low Risk	–0.222		***	0.002	–0.123	*	0.085	–0.164	***	0.005
Medium Risk	0.124		***	0.006	0.088	*	0.087	0.059	*	0.079
High Risk	0.098		***	0.005	0.035		0.117	0.105	***	0.009

(ref: working in the usual workplace)

^***^ Statistical significance: 1%; ** Statistical significance: 5%; * Statistical significance: 10%

The marginal effects of being furloughed due to the pandemic are lower though significant. This transition reduces the probability of being at low-risk, more so for men than for women (29.8% vs. 11.5%) and increases the likelihood of being at medium and high-risk for both men and women. Specifically, this probability is more than double for men in the case of those at high-risk (14.7% versus 6.4%).

Transitioning to teleworking also makes a difference to PWB. Again, it reduces the chances of being at low-risk by 12.3% and 16.4% for men and women respectively, while increasing the possibilities of being at high-risk. In this case, women are particularly affected (the probability of having a high risk of worse PWB being 10.5% for women versus 3.5% for men).

Finally, a transition that is not directly linked with the pandemic, but relevant nonetheless, is that of transition from employment into unemployment taking place before the lockdown. This transition negatively affects men’s PWB, with no significant effects for either women or the sample as a whole. The margins values are slightly smaller than those for lockdown-related joblessness.

## Conclusion and Discussion

The analyses carried out in this research had their roots in interest in the potential effects that sudden, unexpected changes in employment may have on psychological well-being. The main advantage of this approach is that the suddenness of these changes reduces the possibility of bidirectional influence between work-related transitions and psychological states. Probably the most important takeaways from the research concern transitions from employment into joblessness. The fact that it was possible to distinguish between unemployment transitions before the pandemic took place and those resulting from the lockdowns is worth highlighting. The reason why this is noteworthy lies in the possibility that said bidirectional influence might be present in the pre-pandemic transitions, which occurred at any time during the 12 months before the lockdowns, but not in the COVID transitions, which originated from a sudden shock. Comparing the marginal effects of these two unemployment transitions vis-à-vis maintaining employment in the workplace, we see that the results for men are rather similar and increase the risk of PWB problems. However, results for women are quite different, with no significant effects of pre-pandemic unemployment transitions on PWB. Thus, the takeaway is that both men and women share similar marginal effects in pandemic unemployment transitions, confirming the negative effect of joblessness on PWB.^5^

The difference in the effects of pandemic unemployment transitions on men and women deserves further discussion. Firstly, there is ample evidence of the greater psychological vulnerability of women compared to men during the COVID pandemic, and different authors have stressed the role played by different factors: an overload of care tasks in a context of unequal distribution of family duties and chores including childcare (Adams-Prassl et al, 2020; Sevilla & Smith, 2020), a greater fear of contagion (Oreffice & Quintana-Domeque, 2021), concerns regarding domestic violence and family stress from confinement (Béland et al., 2021) or the impact of reduced social interaction (Etheridge & Spantig, 2020). The conclusions of this research support this idea. Results from the ordered probit models by gender conclude that the presence in the household of people more likely to need a greater degree of care (people at special risk from COVID-19 or minors) is associated with a worsening of PWB in women, but not men. The probit model carried out for the whole sample shows that, all things considered, women still bore a greater PWB burden during the lockdown, which fits with the available evidence on the added exhaustion of women, irrespective of whether they work from home (see Meyer et al. (2021), for an analysis in Germany) or in the workplace (see Rodríguez-López et al. (2021) for a sectoral case in Spain). Thus, our results coincide with the general conclusions found in the literature which highlight the negative differential for women in terms of pandemic PWB effects.

Secondly, the results obtained regarding work transitions into furloughs and teleworking are noteworthy, and gender differences stand out. For those involved in both types of transition there are increased risks of worsening PWB when compared with those maintaining their jobs in the usual workplace. Being furloughed has less of a negative impact on PWB than becoming unemployed, a result that is consistent in all of the models. Teleworking also presents negative impacts that are less severe than either becoming unemployed or being on furlough. However, when the analysis is carried out for women only, the estimations of marginal effects show that teleworking produces a greater negative effect on PWB than being on furlough (a 10.5% vs. 6.2% increased probability of becoming at high-risk). This result would be striking during labour market disruption different to that caused by COVID-19. What makes the pandemic unique, among many other factors, is the combination of work disruptions and increased family duties. Thus, our results are in keeping with the analyses carried out in Japan, where mothers have shouldered the burden of working remotely as well as taking responsibility for most of the care of small children at home (Yamamura & Tsustsui, 2021). They also coincide with the German study by Meyer et al. (2021) which highlights the exhaustion of women who work from home when childcare is unavailable. School closures during the lockdowns have thus contributed to the labour market effects of the crisis in PWB, a point made also by (Farre et al, 2021) for Spain, thus contributing to the more severe effects on women than men.

Thirdly, while results for men confirmed the expected patterns regarding the unhealthy effects of restrictive confinements, results for women do not. The probit model corresponding to women shows that living in countries with more stringent restrictions is associated with a reduced probability of worsening PWB when compared to residing in areas without restrictions. These results could be related to a greater fear of contagion, and thus better PWB in the presence of restrictive measures which could reduce the probability of infection. Another possible explanation for this could be that since women are especially concerned about people at risk of COVID-19 and minors in their homes, it may be reasonable to conclude that, in this pandemic situation, they sacrifice freedom of movement as long as the confinement situation protects their at-risk relatives.

In summary, it appears that the COVID-19 pandemic and the measures to control it have worsened the PWB of women to a much greater extent than that of men. Regarding employment transitions, this greater susceptibility is most notable among women who have become unemployed since the pandemic started and those who telework. Furthermore, in view of the results contained in this work and the reviewed bibliography, these differences seem to be related to the concurrence of labour market disruption and increased family duties, which are born less by men than women.

This research is not, however, without limitations. First, although the exogenous shock created by confinement made it possible to minimize the bidirectionality between the variables of employment situation and PWB, that is, the probability that a person is unemployed, furloughed or teleworking due to a specific PWB state, the nature of the variables does not allow us to rule out the possibility of bidirectionality entirely. Furthermore, the way of obtaining the data, through an online survey, could have introduced some bias such as, for example, absence or scarcity in the sample of people without access to technology, or those unfamiliar with this type of data collection method.

Regarding future research, it is expected that the availability of longitudinal data will allow us to exploit additional records about employment transitions and PWB, thus permitting a greater reduction in the aforementioned endogeneity of employment situation variables.

## Data Availability

Not applicable.

## Footnotes

1 Using the score obtained in the General Health Questionnaire (GHQ-12) as the dependent variable, the concept of PWB is defined in this paper as the degree to which a person shows symptoms of non-psychotic morbidity (Goldberg, 1967), mainly anxiety and depression (Kiliç et al, 1997). The concept of PWB, used to refer to the score obtained in the GHQ-12, has already been used by other authors (Sánchez-López & Dresch, 2008).

2 The complete list of countries is Andorra, Argentina, Australia, Belgium, Belize, Cambodia, Canada, Chile, Colombia, Denmark, Ecuador, France, Germany, Hungary, Italy, Japan, Jordan, Mexico, Morocco, New Zealand, Poland, Portugal, South Africa, Spain, Sweden, Switzerland, Turkey, United Kingdom and the United States of America.

3 The weighting coefficients have been obtained from the sample of the last Spanish National Health Survey, carried out in 2017.

4 It is possible to assume other distributions for *ε* such as, for example, the logistic one.

5 If we were to assume bidirectional effects between PWB and work-related transitions, these would pertain to men only.
